# Case Report on Caries Assessment Using Intraoral Scanner Compared with Bitewing Radiographs

**DOI:** 10.1055/s-0044-1782192

**Published:** 2024-05-02

**Authors:** Bernard Siew, Joachim Enax, Frederic Meyer

**Affiliations:** 1 Smilefocus, Camden Medical Centre, Singapore, Singapore; 2 Research Department, Dr. Kurt Wolff GmbH & Co. KG, Bielefeld, Germany

**Keywords:** dental caries, near-infrared light, NIRI, bitewing radiograph, toothpaste, hydroxyapatite, digital dentistry

## Abstract

Dental caries remains one of the main reasons patients seeing their dentist. They either show up for preventive measures, diagnostics, or treatment of present caries lesions. To date, diagnostics are performed visually, supported by using bitewing radiographs. While radiographic diagnostics should only be performed on a biannual basis, and some caries process will not be seen with visual diagnostics, there remains a lack in regular checkups. Therefore, different technical applications can be used for regular diagnostics. One of those is the near-infrared imaging (NIRI) technology. In this case report, a patient presented with incipient caries lesions. These lesions were diagnosed visually, radiographically, and using NIRI. After diagnosis of incipient caries lesions, the patient was advised to use a hydroxyapatite toothpaste and a hydroxyapatite gel for the remineralization of the lesions and prevention of caries progression. The patient was followed up for 6 months with regular checkups in between. Visual diagnostics and NIRI were used to detecting the caries progress. After 6 months, bitewing radiographs were used in addition. In this clinical investigation we were able to show for the first time that NIRI and bitewing radiographs are able to detect and follow incipient caries lesions. Additionally, this study highlights that hydroxyapatite-containing oral care products are able to arrest and remineralize the caries process.

## Introduction

The treatment of dental caries remains to be one of the main reason for dental visits. 1 Caries in a patient can vary from mild incipient lesions to rampant and extensive breakdown. 2 Dental caries is caused by the consumption of a carbohydrate- and sugar-rich diet and poor oral hygiene. This stimulates the metabolism and overgrowth of acidogenic bacteria within the dental plaque. The produced acids can subsequently attack the dental hard tissue. 3 4 At the same time, early carious lesions, or incipient lesions, can be remineralized under ideal oral conditions. Patients with incipient lesions can benefit from remineralizing actives used for daily oral care. Recently published clinical trials, literature reviews, a meta-analysis, and various *in vitro* investigations have shown that toothpaste containing biomimetic hydroxyapatite inhibits the progression of dental caries comparable to the use of fluoridated toothpaste. 5 6 7 8 9 10 11 Unlike fluorides, the use of biomimetic hydroxyapatite is not limited by a cytotoxic threshold and may thus be applied to the teeth several times a day in very caries-active patients. 12 13 Following this, products with hydroxyapatite can be applied several times a day. Additionally, studies have shown that hydroxyapatite leads to a homogenous remineralization to even deep layers. 8 Thus, hydroxyapatite seems to be a highly suitable active ingredient for daily oral care and especially with patients suffering from incipient lesions.

Nevertheless, patients with initial lesions need to be routinely and closely monitored, as dental caries can progress to deeper cavities. Currently, periodic bitewing radiography is used to monitor the control of the caries progress. 14 One major limitation of bitewing radiographs is the exposition to X-ray. Even though modern radiographic imaging machines are able to minimize irradiation (0.01 mSv), the use of bitewing radiographs is advised to be limited as for the safety of the patients and the dental workers alike. 15 Therefore, scanning devices using different technologies have been developed. One of those is near-infrared imaging (NIRI). 16 The use of NIRI is employed with a three-dimensional (3D) scanner, iTero 5G. A few studies have been published showing that this device might be suitable at detecting caries lesions under *in vivo* conditions. 16 17 18 In a clinical study with 100 patients Metzger et al analyzed NIRI technology in comparison to bitewing radiographs for detecting proximal caries. 16 They found that early caries lesions can be detected with an accuracy of 88% and established caries can be detected with an accuracy of 97%. Additional to this clinical trial, Litzenburger et al and Schlenz et al analyzed this technique in *in vitro* studies. 17 18

Here, we describe the case of a patient using a hydroxyapatite regime for the remineralization of initial caries lesions. For the monitoring of the dental caries lesions, NIRI was used with every dental checkup, and bitewing radiographs were used every 6 months. The aim of this setup was to compare the efficacy of the hydroxyapatite regime and the accuracy of NIRI technology compared with bitewing radiographs.

## Case Report

**Fig. 1:**
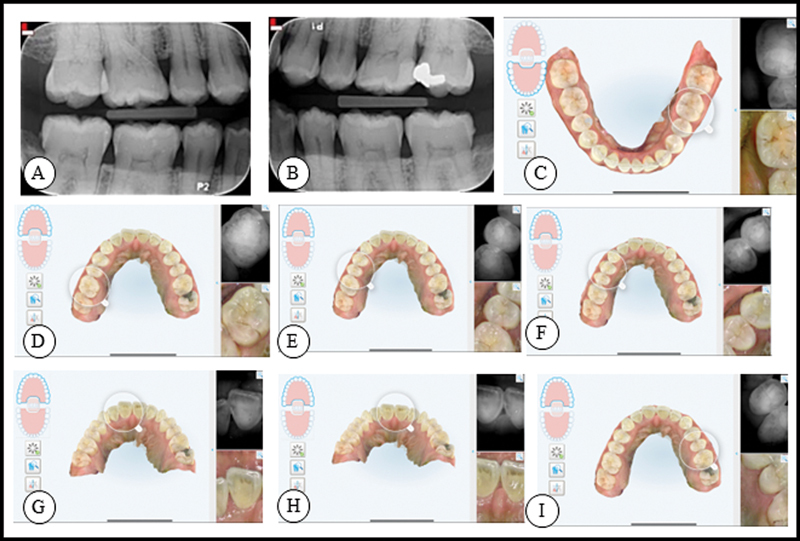
( **A** ) and ( **B** ) show the bitewing radiographs taken from the patient at the baseline visit. ( **C** ) to ( **I** ) represent NIRI images that were made using iTero scanner of teeth #35 and #36 ( **C** ), #16 ( **D** ), #14 and #15 ( **E** ), #14 ( **F** ), #11 ( **G** ), #11 and #21 ( **H** ), and #24 and #25 ( **I** ). NIRI, near-infrared imaging.

A 41-year-old female patient with an interest in tooth correction using the Invisalign technology presented with a history of minor dental restorations, good-to-moderate oral hygiene, and several dental incipient lesions. Her plaque score at each visit was, depending on the tooth, between 0.5 and 2.0 according to the Turesky modification of the Quigley and Hein Plaque Index. 19 Fig. 1 presents the dental status after bitewing radiographs and live images using NIRI and iTero 3D scan (Align Technology Inc., Tempe, Arizona, United States) were taken. The patient had incipient caries detected on the following teeth: #16, #15, #14, #11, #21, #24, #25, #36, #35, and #45. The caries detection was also verified on the bitewings and clinically observable.

The patient reported that her diet did not contain sugar-rich food or intake of erosive foods and drinks. She did not have any diet restrictions or allergies. Her weekly alcohol and sugar intake was considered moderate. She reported no hyposalivation or dry mouth. Her home care routine included the use of an electric toothbrush with fluoride toothpaste (>1,000 ppm F ^−^ ) twice a day. However, incipient caries lesions developed. In the following, the patient was advised to brush twice daily with a hydroxyapatite-containing toothpaste (Bioniq Repair-toothpaste, Dr. Kurt Wolff GmbH & Co. KG, Bielefeld, Germany) and application of a hydroxyapatite-containing gel (Karex tooth protection gel, Dr. Kurt Wolff GmbH & Co. KG, Bielefeld, Germany) overnight. The gel was directly applied on the teeth. She was advised not to alter her regular diet or lifestyle.

The patient was then clinically observed for physical changes on the dentition. No scans were taken at the first follow-up after 1 month. At the follow-up after 2 months, a scan was taken, and clinical observation of the caries lesions was performed.

**Fig. 2:**
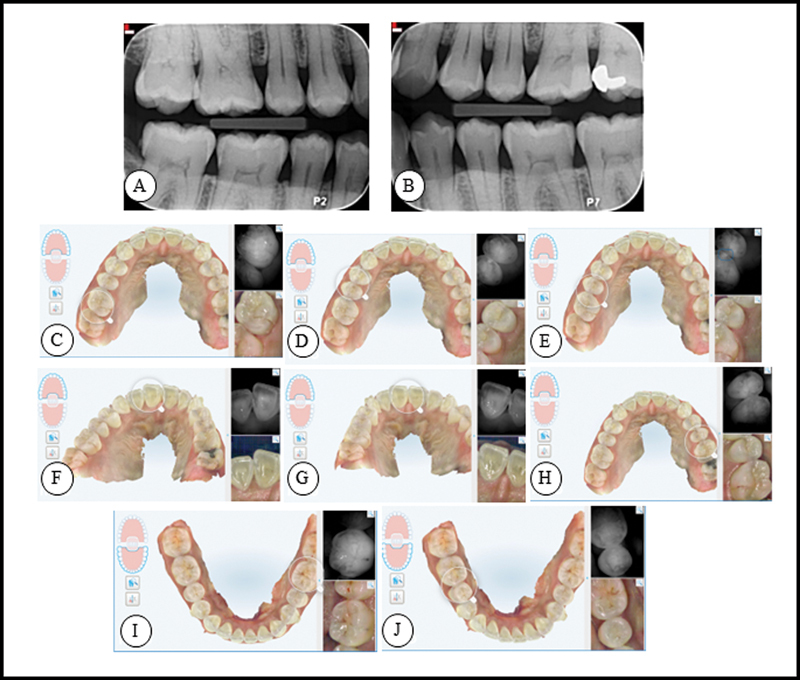
**A** and **B** show the bitewing radiographs taken from the patient after 6 months. **C** to **I** represent NIRI images that were made using iTero scanner of teeth #16 ( **C** ), #15 ( **D** ), #14 ( **E** ), #11 ( **F** ), #21 ( **G** ), #24 ( **H** ), #25 ( **I** ), #36 and #35 ( **J** ) after 6 months using a hydroxyapatite toothpaste and a hydroxyapatite containing gel. NIRI, near-infrared imaging.

Fig. 2 shows the results after 6 months.

**Fig. 3:**
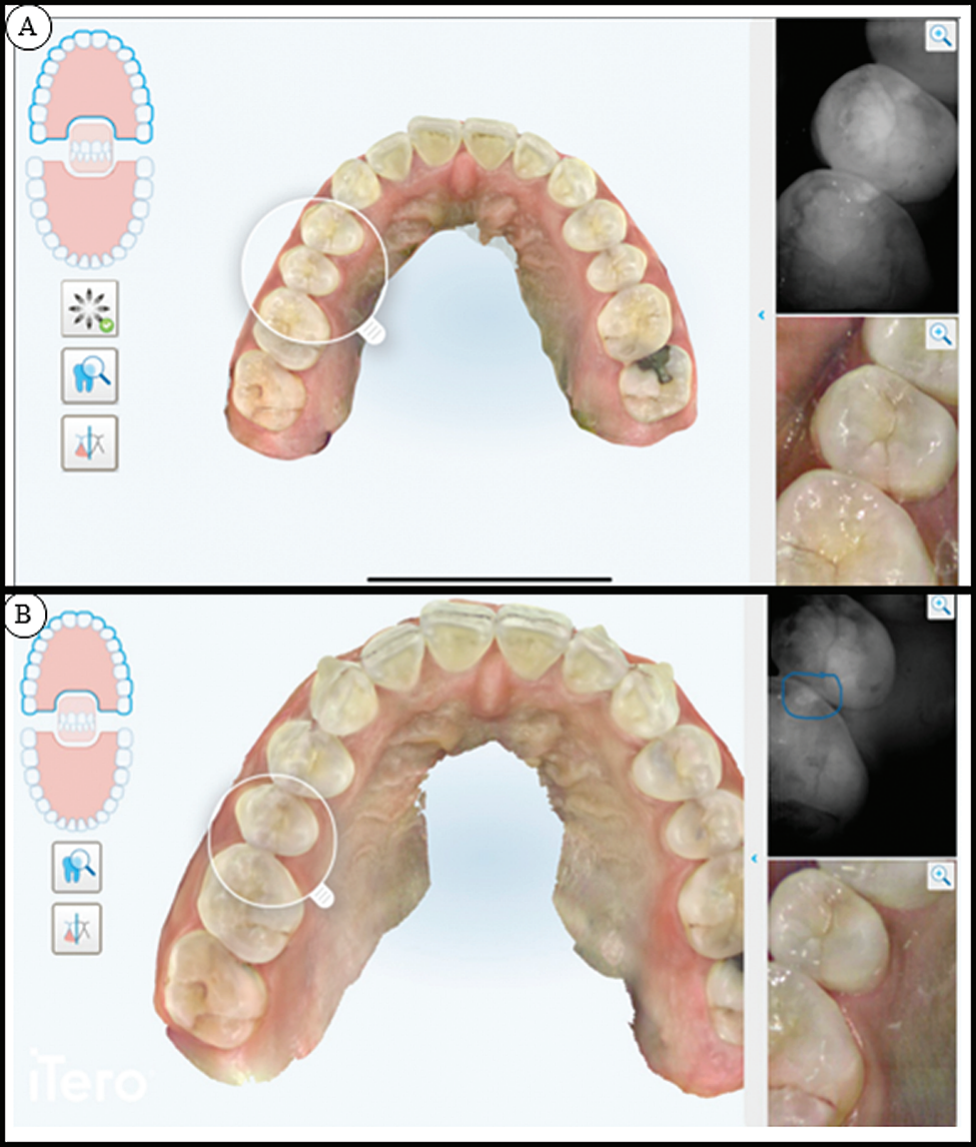
Teeth #14 and #15 at baseline and after 6 months. The iTero scans and NIRI are able to show the lesions. After 6 months, an improvement of the lesions is clearly visible. NIRI, near-infrared imaging.

The bitewings and scans show a consistent result of caries status. Fig. 3 shows the comparison at baseline ( **A** ) and after 6 months ( **B** ) using NIRI and iTero scans of teeth #14 and #15. Plaque scores remain the same, between 0.5 and 1.5. Notably, the occlusal surface of tooth #36 appeared to have hardened when examined clinically. The stained pit also appeared less “wet” during visual and tactile diagnosis. The surface of this tooth felt smooth to the probe. It is important to note that this carious lesion was not indicated on the bitewing radiographs nor on NIRI. Bitewing radiographs, NIRI, and visual/tactile diagnostic showed consistent results. Hydroxyapatite-containing oral care products stopped the caries progress consistently.

## Discussion

The dental caries status in the subject improved to arrested and partially remineralized. Hydroxyapatite has been shown to effectively remineralize and prevent dental caries in children, 9 adults, 7 and high-risk patients with orthodontic appliances. 6 10 20

Dental caries has been shown to be mainly caused by cariogenic diet and poor oral hygiene habits, where dental plaque removal is not sufficient. 4 However, dental caries can also occur, when oral hygiene habits and dental plaque removal is good. Nevertheless, it needs to be mentioned that even in the absence of visible plaque or stainable dental plaque, bacteria may still be present on the tooth surface that are hard to reach with a toothbrush, such as fissures and interproximal lesions. This can be seen in this case, as the plaque index is between 0.5 and 1.5, depending on the investigated tooth, which is indicated to be low. 19 While the patient used a fluoride toothpaste to prevent caries, 21 she still developed incipient caries. This clearly shows that prevention of dental caries cannot follow a “one-size-fits-all” concept using fluorides. Here, the use of hydroxyapatite-containing oral care products led to the remineralization and prevention of dental caries lesions, which is consistent with the existing literature. 6 7 8 13 20 22 23 24 25 26 Furthermore, compliance of the patient regarding the respective oral care products is of high importance. This is the same for the use of digital scanning technologies or the use of radiographs.

Another aspect of this case report was to assess the reliability of NIRI compared with bitewing radiographs. In contrast to bitewing radiographs that are using low levels of X-ray, NIRI is based on near-infrared light technology. NIRI is used as X-ray is known to cause further health problems in case of constant exposure. 15 In addition to NIRI, a 3D scan was used to visualize the defects of the enamel and dentin. 16 17 18 The results found here go in line with the *in vivo* study on the accuracy of this tool that have been previously published. 16 However, both technologies need to be combined with visual and tactile diagnostics, as at least one lesion in this study could not be observed either with NIRI or bitewing radiograph. However, the 3D scan with the NIRI were useful tools to assist with the assessment and the record of the incipient caries lesions.

## Conclusion

This case report points out that there is consistent evidence on hydroxyapatite-containing toothpaste and gel to effectively and predictable remineralize incipient caries. In addition, NIRI was found to assist measuring the remineralization process but does not outperform bitewing radiographs. However, NIRI can be performed with every dental visit and the results on measuring remineralization are promising, especially on carious lesions of occlusal surfaces. Patient compliance is the key for the use of oral care products or the use of digital imaging technologies. Future studies are needed to test if NIRI can also measure remineralization using different remineralizing agents, such as other calcium phosphates or fluoride. It must be concluded that demineralized tissue can only be assessed as active or arrested/remineralized after an oral examination by a dental clinician. The iTero scans in combination with NIRI were found to be very useful as a visualization tool. NIRI, in addition, was useful to indicate interproximal lesions similar to conventional transillumination with the added advantage of the digital record. Following the results from this case report, it is promising to consider that dental clinicians can systematically and periodically scan their patients' teeth to monitor progress on oral hygiene, teeth alignment, teeth wear, interproximal, and surface caries without excessive use of dental irradiation images.
